# Applying neural networks to predict HPC-I/O bandwidth over seismic data on lustre file system for ExSeisDat

**DOI:** 10.1007/s10586-021-03347-8

**Published:** 2021-07-02

**Authors:** Abdul Jabbar Saeed Tipu, Padraig Ó Conbhuí, Enda Howley

**Affiliations:** 1 School of Computer Science, National University of Ireland Galway, Galway, Ireland; 2 Irish Centre for High-End Computing, Dublin, Ireland

**Keywords:** ExSeisDat, HPC, MPI-I/O, SEG-Y, Lustre file system, Machine learning, ANN, MSE, MAE, MAPE

## Abstract

HPC or super-computing clusters are designed for executing computationally intensive operations that typically involve large scale I/O operations. This most commonly involves using a standard MPI library implemented in C/C++. The MPI-I/O performance in HPC clusters tends to vary significantly over a range of configuration parameters that are generally not taken into account by the algorithm. It is commonly left to individual practitioners to optimise I/O on a case by case basis at code level. This can often lead to a range of unforeseen outcomes. The ExSeisDat utility is built on top of the native MPI-I/O library comprising of Parallel I/O and Workflow Libraries to process seismic data encapsulated in SEG-Y file format. The SEG-Y File data structure is complex in nature, due to the alternative arrangement of trace header and trace data. Its size scales to petabytes and the chances of I/O performance degradation are further increased by ExSeisDat. This research paper presents a novel study of the changing I/O performance in terms of bandwidth, with the use of parallel plots against various MPI-I/O, Lustre (Parallel) File System and SEG-Y File parameters. Another novel aspect of this research is the predictive modelling of MPI-I/O behaviour over SEG-Y File benchmarks using Artificial Neural Networks (ANNs). The accuracy ranges from 62.5% to 96.5% over the set of trained ANN models. The computed Mean Square Error (MSE), Mean Absolute Error (MAE) and Mean Absolute Percentage Error (MAPE) values further support the generalisation of the prediction models. This paper demonstrates that by using our ANNs prediction technique, the configurations can be tuned beforehand to avoid poor I/O performance.

## Introduction

**Fig. 1:**
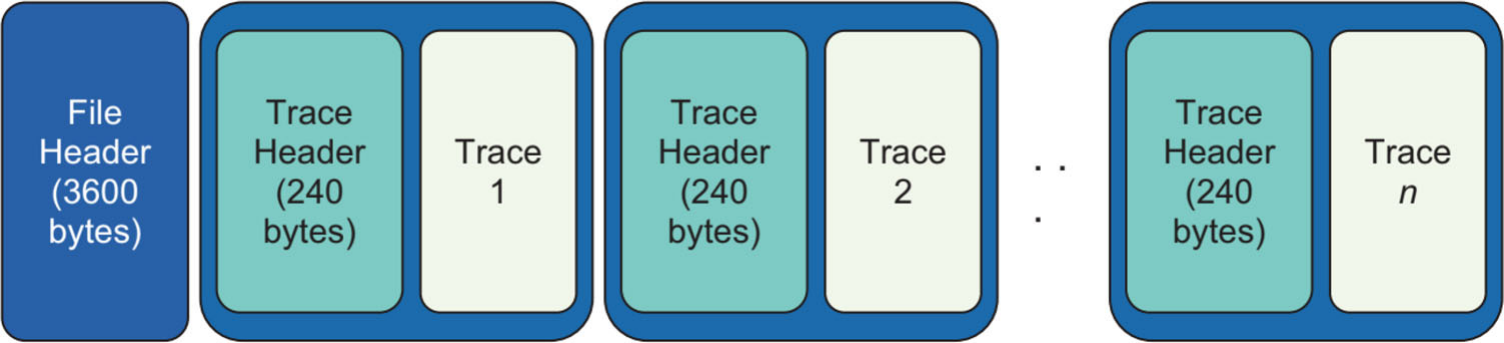
Structure of SEG-Y file format

Seismic data is one of the most critical factors for geophysicists to study and understand the earth structure beneath its surface or seabed. Aside from being of critical importance in understanding our globe and when earthquakes and tremors might jeopardise human life [1], the study of Seismic data is also a critical factor for the Oil and Gas industry [2]. The SEG-Y File format is the standard across the globe for the encapsulation and processing of the seismic data [3]. The SEG-Y File format shown in Fig. 1 is typically very complex with alternative arrangements of traces, preceded by their corresponding headers. Its is also quite common for its file size to reach petabytes in scale. This dramatically increases demands on high performance based I/O processing of seismic data across the research and Oil/Gas production industry. This is where the High Performance Computing (HPC) or super-computing clusters play a significant role.

The Extreme-Scale Seismic Data (ExSeisDat) Library is developed to process the SEG-Y files efficiently by further using its Parallel-I/O Library (PIOL) and Workflow Library on the HPC clusters [4]. It is based on the C++ language platform and the standard Message Passing Interface (MPI) Library, a parallel-distributed memory framework, which provides a large set of Application Interfaces (APIs) to exploit the potential of parallelism within the clusters [5]. Commonly, the parallel MPI-I/O struggles in overcoming the performance degradation of a program because as it relies on certain parameters to project the I/O bandwidth. This is also the case with respect to MPI-I/O when applied to ExSeisDat data and the processing of SEG-Y files. These important parameters are related to a number of components employed on clusters. For example, the number of MPI processes running on compute nodes, the Parallel File System (PFS) managing multiple storage objects which is known as the Lustre File System (LFS) ( [6]) in our case, and also the file properties, access patterns, etc.

Previous research has shown that I/O performance prediction based on the different parameters settings can result in significant benefits [7–11]. Despite the key differences between this and existing research with respect to parameters and ML techniques, the prediction of SEG-Y file I/O performance beforehand is itself innovative. It can be immensely beneficial for tuning the related configuration parameter settings to overcome the poor I/O performance during the execution of MPI application. In [12], the Artificial Neural Networks (ANN [13, 14]) based approach was used as a ML technique to estimate the I/O performance based on the file access times and patterns and showed 30% less prediction error in comparison to linear models. This provided the motivation for us to apply ANNs to this problem, arising from its forecasting capabilities to the key input parameters, and predict the I/O bandwidth before execution of the actual I/O operation within application or program. The high prediction accuracy of Deep Learning (DL) ANNs has also been proven on different frameworks and applications as mentioned in [15]. The PyTorch is one of those DL frameworks we have used for developing and training the ANN models [16].

The critical idea is to use the predicted bandwidth as a means of optimizing the I/O performance by tuning the parameters. The tuning of parameters can be those settings from the evaluations of benchmarks execution results, given later in this paper. In addition to this, some can be the combinations suggested in previous research that can increase the I/O bandwidth performance on the LFS based cluster in [17–20]. These existing studies suggest that by setting the number of MPI processes to the number of parallel hard disks, and chunk size (being read or written by each MPI process) to stripe size (file striping unit over hard disks in round robin fashion) can significantly increase the I/O bandwidth performance. There are another series of approaches that are outside the scope of this paper. These include manipulating Remote Procedure Call (RPC) thread counts, controlling the Object Storage Targets (OSTs or hard disks) and using the single MPI calls to read or write large chunk sized *MPI_Datatype* [21, 22].

Our research focuses primarily on the very basic and useful parameter settings that can be considered for the prediction of bandwidth values. These settings can enhance I/O throughput, as shown with the support of parallel HiPlot utility’s graph plots ( [23]), from separately executed MPI-I/O, SEG-Y File I/O and Sorting benchmarks from ExSeisDat. These parallel plots of I/O bandwidth values against different parameters settings is another novelty of this research. In addition to this, the other primary focus of our approach is the prediction of I/O bandwidth prior to its runtime, and therefore potentially yielding significant performance outcomes in terms of accuracy.

The prediction results show that our ANN based models are sufficiently generalized to predict the I/O bandwidth behaviour. This paper is structured as follows; Related Work in Sect. 2, Design and Implementation in 3, Experimental Result Analysis in 4, Discussion in 5 and Conclusion in 6.

## Related work

Previous research has examined where I/O behaviour was machine-observed using certain ML techniques, parameters and environment, subsequently prediction of I/O was used as a tool to overcome poor performance in I/O bandwidth. These research studies motivated us to approach the prediction of I/O performance for ExSeisDat over SEG-Y format files distributed over LFS based networked storage in HPC clusters.

In [7], it has been demonstrated that HPC I/O is affected by factors like CPU frequency, number of I/O threads, and the I/O scheduler. The I/O behaviour is predicted and determined on the basis of these factors using interpolation and extrapolation techniques by developing a model using a data analytic framework over large-scale experiments. The performance of the methods is being evaluated by measuring prediction accuracy at previously unseen system configurations. Then the methodology for optimizing system configurations uses the estimated variability map based on Bayesian Treed Gaussian Process and some other regression methods. This yield new insights into existing statistical methods for the practice of HPC variability management in terms of parameters selection.

The work presented in [8], is the adaptive method to schedule parallel I/O request for any application on any HPC system by tuning the parameters depending on time window of current workload. The adaptive method is formulated using reinforcement learning of the scheduling algorithm. It achieves 88% of precision to select parameters on runtime after observing the access pattern (contiguous or non-contiguous) for few minutes. Consequently, the system will be able to optimize its I/O performance for rest of its life, as claimed.

The approach presented in [10], is the random forest regression modelling, used as the ML technique to predict the I/O bandwidth for only collective write operation in MPI-I/O Library. The accuracy of prediction is very high. It varies from 82 to 99% approximately depending upon maximum depth setting, but the training and testing data sets are very small in comparison to the size of our benchmarks data sets. The accuracy can be lower if more variation is added in data sets. The remaining differences are the format of bench-marking file, the parameters and the processor or cluster hardware.

In [9], the I/O optimization is proposed for HDF5 file format parallel applications on various HPC platforms with LFS and GPFS. The parallel I/O is optimized via auto-tuning, supported by I/O modeling of Lustre’s IOR (Interleaved or Random) benchmarks and other I/O benchmarks, through nonlinear regression models prediction. The tuning achieves significant increase in I/O bandwidth for different applications on the HPC platforms, by means of prediction and selection of new parameters values. To some extent, [11] also provides the parallel I/O predictive modelling of LFS IOR benchmarks by developing a Gaussian process regression ML model.

The research presented by Schmidt et al. in [12], predicts the file access times on a Lustre file system from the client end. File access times are measured in various test series and are then used to develop different models to predict them. The evaluation shows that these models which utilize ANNs, give 30% less average prediction error in comparison to linear models. The distribution of file access times was evaluated with respect to file accesses using identical parameters. The typical access times usually differ by orders of magnitude and depend upon an alternative I/O path.

In addition to this, few other researches are also explored in the context of prediction based optimization for MPI applications through ML and auto-tuning parameters, but they all lack consideration of the I/O side [24, 25].

In contrast to these existing studies, our research approach focuses on predictive modelling of I/O bandwidth, using ANNs, over seismic data in the form of SEG-Y File format as our primary usecase for this paper.

## Design and implementation

### Research methodology

**Fig. 2:**
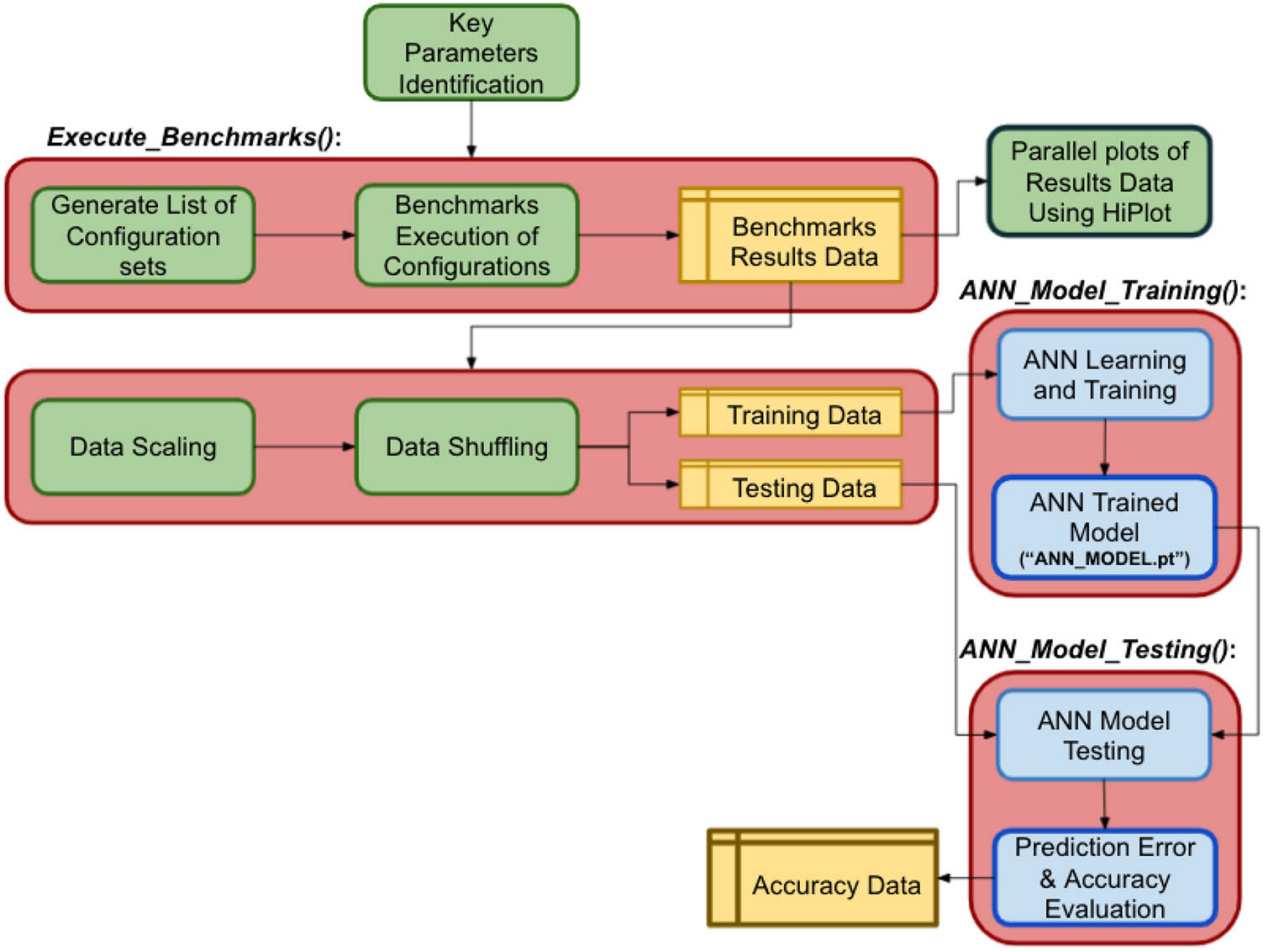
Research flow

This research is conducted in number of steps which are: (1) Identification of key configuration parameters, (2) Generating list of configurations values sets, (3) Development of Benchmarks over key parameters, (4) Execution of Benchmarks over key parameters, (5) Collection of runtime performance data as a training set for ML, (6) Training of ANN model over collected training set, (7) Prediction of I/O performance over test set (20% of collected data) and (8) Accuracy evaluation for predicted results. Figure 2 shows the main components of our research model.

### Key parameters identified

In this research, three types of benchmarks from ExSeisDat are executed: (1) basic MPI-I/O benchmarks to read and write stream of bytes in file, (2) SEG-Y File I/O benchmarks to read and write seismic trace data and (3) SEG-Y File sorting benchmarks to sort seismic trace data from different unsorted orders. It should be noted that some parameters are overlapping for all benchmarks and some are different due to the nature of their workings which are explained in later sections.

Table 1 shows the complete list of key parameters identified and the values used to conduct each of the three benchmarks. The most right column of this table has been merged to show that both (SEG-Y I/O and Sorting) benchmarks have the same parameters except some of their different possible values which are explained later.

**Table 1:** Configuration parameters and their values

Parameters	Benchmarks	
	MPI-I/O	SEG-Y I/O & Sorting
Number of MPI nodes	2,4,8,16	2,4,8,16
MPI processes per node	1,2,4,8	1,2,4,8
Stripe count	2,4,8,16	2,4,8,16
Stripe size (MBs)	1,256,512,1024,2048	1,256,512,1024,2048
File size (GBs)	1,2,4,8,16,32	–
Chunk size (GBs) per process	0.25,0.5,1,2	–
Io operation	read/write	Read/write
File access pattern	Collective/non-collective	Contiguous/random
Number of traces	–	512,1024,2048,4096
Samples per trace	–	256,512,1024,2048
Unsorted order	–	Uniform/reverse/random

### Development of benchmarks

This Section explains working of each benchmark type, implementation and formulation of results.

#### Working of MPI-I/O benchmarks

MPI-I/O benchmarks are the simplest, such that MPI processes on each node would perform READ or WRITE operations on their respective chunk sizes in a single file size striped across number of Lustre disks (stripe count) with a certain stripe size. The MPI read or write calls would be either *collective* (where each process also accesses neighbouring processes memory space) or *non-collective* (where each process accesses only their own memory space) to complete the parallel I/O. Considering all the possible values mentioned in Table 1 from MPI-I/O benchmarks, the total number of possible configurations of parameters or the total number of these benchmark executions would be 30720 (15360 for each READ and WRITE operations).

#### Working of SEG-Y File-I/O benchmarks

SEG-Y File-I/O Benchmarks are different than basic MPI-I/O benchmarks. In this case, they are reading or writing (modifying) a combination of traces and samples per trace within an already generated SEG-Y file with only *uniform* order. MPI processes in these benchmarks would be either read or write using *contiguous* or *random* access patterns as can be seen from SEG-Y File-I/O Benchmark parameters stated in Table 1. As the SEG-Y file is Lustre striped over a number of OSTs, therefore the number of traces and samples per trace are distributed over different disks with a certain stripe size. In this case, the total possible configuration of parameters or executions of this benchmark type are 20480 (10240 for each READ and WRITE operations).

#### Working of SEG-Y file sorting benchmarks

Sorting Benchmarks are more complex than the SEG-Y File-I/O benchmarks. In these benchmarks, first a SEG-Y file would be generated with any of the unsorted orders: (1) *uniform*, means SEG-Y file is sorted in ascending order with respect to source-x coordinate from the trace header value [3], (2) *uniform_reverse*, means trace data is arranged in descending order with respect to source-x or (3) *random*, means trace data is arranged in arbitrary manner, as mentioned in Table 1. Then MPI processes in each sorting benchmark would either read or write with only contiguous access pattern the number of traces and samples per trace in SEG-Y file, Lustre striped. Therefore, in this scenario, the total possible configuration of parameters or executions of this benchmark type are 30720 (15360 for each READ and WRITE operations).

#### Code structure generating benchmarks data

**Fig. 3:**
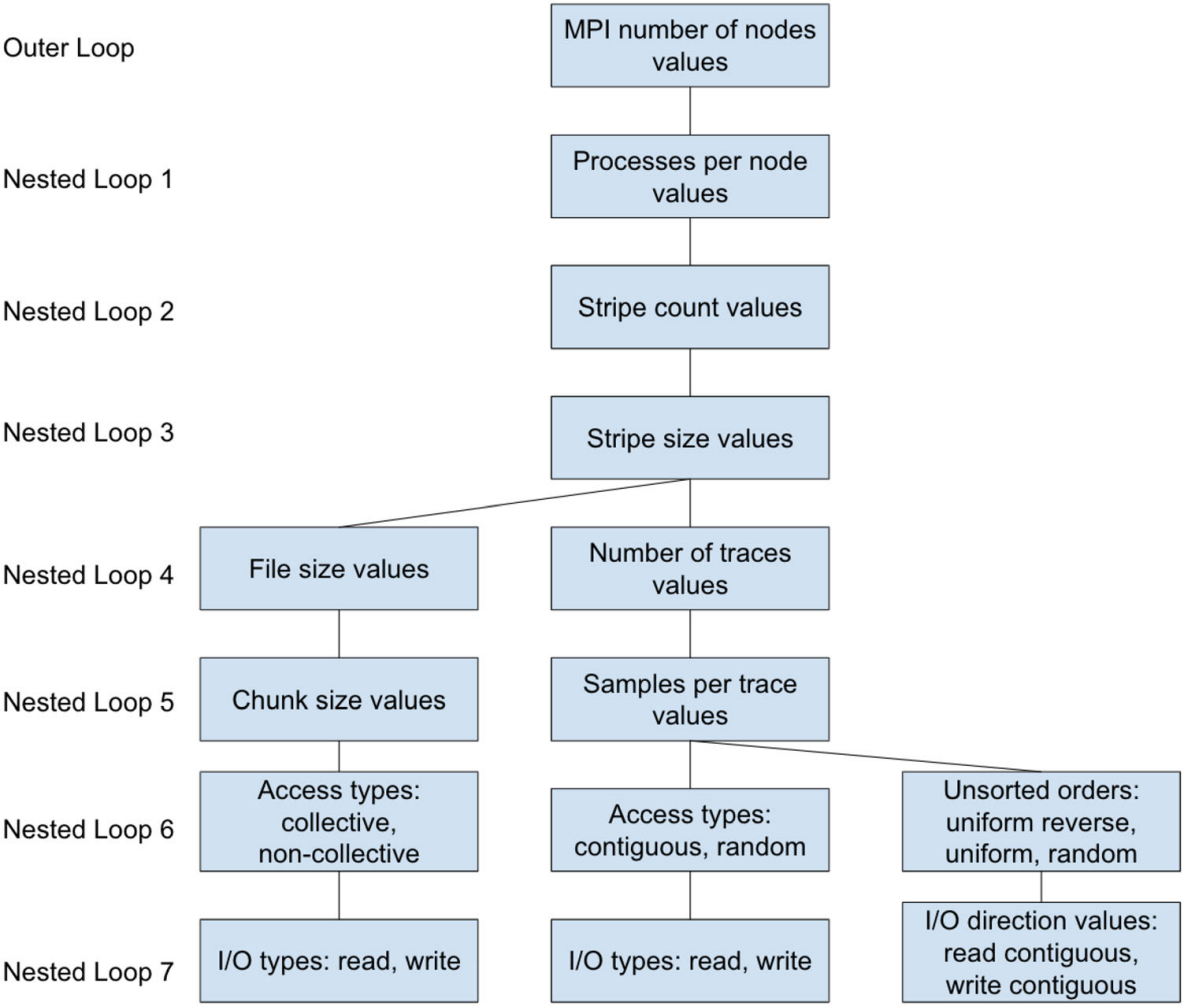
Hierarchy of parameter values nested loops generating all possible configurations to benchmark

The Listing 1, represents the overview of the method to collect I/O bandwidth data against different parameters and values. The parameters and values from Table 1 are passed as arguments to a function definition *Execute_Benchmarks()*, for executing all possible the benchmarks on line 1 in Listing 1. Initially, they are being passed to a function *GenerateConfigsValuesList()* on line 2. This function is responsible to generate a complete proper possible lists of configuration settings according to hierarchy of nested loops for each parameter in Fig. 3. The configurations lists have been generated with respect to each benchmark type (Basic MPI-I/O, SEG-Y File-I/O and SEG-Y File Sorting). The last nested loop completes one set of configuration values which is appended to the list that eventually builds a complete list of all possible configurations for each benchmark type. These lists for each benchmark type are being fetched in a sequence on line 3 with outer-loop. In line 4 with inner-loop a unique set of a configuration setting (*config*) is being fetched from a current benchmark type configurations list (*configs_list*). This one set of configuration setting object represents one execution benchmark for a file i.e. [*MPI_nodes = 16, processes_per_node = 8, stripe_count = 8, stripe_size = 256, io_operation = WRITE, access_pattern = RANDOM, traces = 4096, samples_per_trace = 1024,...*] for one SEG-Y WRITE operation. Apart from configuration settings values in *config* object, it also contains all other necessary information related to currently executing benchmark i.e. benchmark type, input/output file names, the target Darshan profiling file path and name, etc.

For each benchmark execution we are applying pre-benchmark settings for the Lustre File System and Darshan utility on line 11 and 15, respectively. Darshan is a HPC I/O characterization tool, designed to capture I/O bandwidth performance [26]. As discussed previously, benchmarking is completed using Lustre File System disks therefore, before each execution of the benchmarks with certain configurations, the Lustre Striping on target file is executed to distribute the file parts on OSTs. This is being done to test the performance of a particular benchmark configuration with respect to its file striping.

On line 18, an input file is always being generated before the execution of actual I/O benchmark except the MPI-I/O WRITE benchmark. The I/O bandwidth value is recorded by the Darshan utility, once the benchmark is executed over a set of configuration parameters, on line 21, by reading and parsing the generated Darshan file. After the execution of a benchmark the file is being deleted in order to remove it from cache, as a post-benchmark setting on line 25.

#### Formation of results

The results of each benchmark along with their configuration parameters are appended in the YAML file on line 22 of Listing 1. Therefore, each *(config, io_bandwidth)* object written to file is a dictionary object with its *(key, value)* pairs to represent each execution.

### Development of ML based ANN models

In this section, we will outline the approach used to learn and predict the performance behaviour arising from the benchmark results and types outlined previously.

For this purpose, the ANNs have been used to learn the changing performance behaviour over the various configurations on each benchmark’s result.
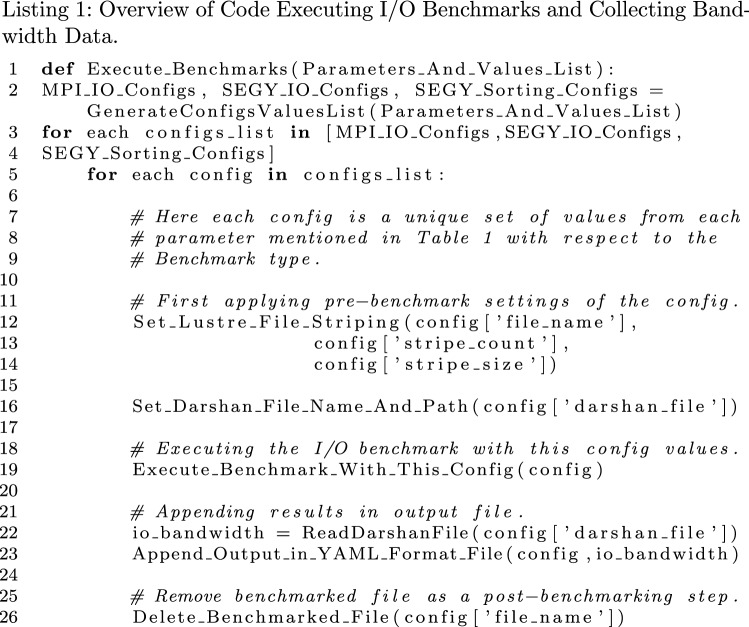


The Listing 2 shows the very basic algorithmic view of the ML process under the definition of function *ANN_Model_Training()*. The formation of ANN’s layers plays a key role for our Machine Learning approach as shown from the line 5 to 14 of Listing 2. The ANN for each benchmark comprises of a 7 node input layer which represents the input parameters, then the output layer has one node to represent a single output as bandwidth value for each input of configuration parameters values. The number of hidden layers chosen are 2, the number of nodes in the first hidden layer are 256, and the number of nodes in the second hidden layer are 128, as described in Table 2. These values represent our Neural Network architectures of the training models for each benchmark type, running independently for read and write operations.

In Table 3, the hyper-parameters applied to the models during the training are described, which eventually supports in improving the accuracy on the test set. The Dropout percentage rate (*nn.Dropout()*) and Rectified Linear Unit activation function (*nn.ReLU()*) are applied on ANN’s nodes and layers during training on line 9, 11 and 13 [27]. These are the example values to demonstrate training for one model otherwise, they are different in actual as mentioned in 4$^{th}$ column from the left side in Table 3. Similarly, the weight decay value on line 19 is also different for each model, as mentioned in 5$^{th}$ or most right column. The weight decays are applied as means of regularization to avoid over-fitting as much as possible. The learning rate (*lr*) on line 18, is same for all models, as mentioned in 3$^{rd}$ column.

**Table 2:** ANN’s description table

Input layer	Hidden layer 1	Hidden layer 2	Output layer
7	256	128	1

**Table 3:** ANN’s Hyper-parameters

Benchmark	I/O	Learning rate	Dropout	Weight decay
MPI-I/O	READ	0.002	0.0	1$e^{-5}$
	WRITE		0.05	1$e^{-5}$
SEG-Y I/O	READ		0.0	0.0
	WRITE		0.05	0.0
SEG-Y Sort	READ		0.05	1$e^{-5}$
	WRITE		0.0	0.0

The main loop shown on line 23 of Listing 2, is responsible for training the ANN models. The training set is 80% of the complete data for each benchmark type operation. It assumes all the data is scaled between 0 and 1 (in addition to random sampling) on line 20 and 21, and uses the following *MaxAbsScaler* formula:
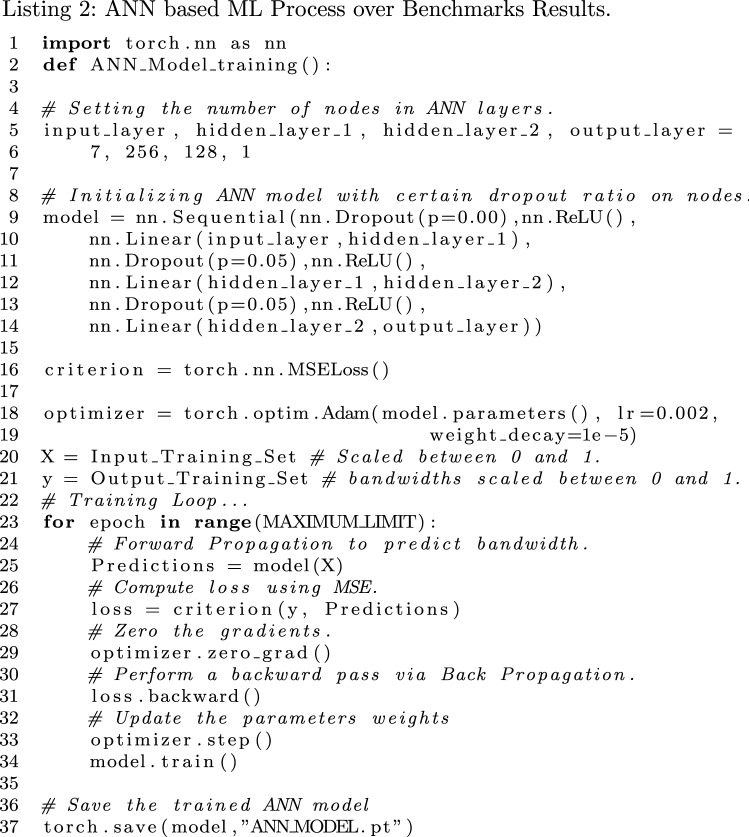

1 $$\begin{aligned} x' = \frac{x}{x_{max}} \end{aligned}$$

where $x'$ is the new scaled value, *x* is the original value and $x_{max}$ is the maximum absolute value of any configuration parameter given in Table 1. For example, as the number of MPI nodes have possible original values (2, 4, 8, 16) therefore, the possible scaled values will be (0.125, 0.25, 0.5, 1.0). The *MaxAbsScaler* is applied on both the input feature parameters (the possible configuration values) and the output feature parameter which is the I/O bandwidth value collected on each configuration benchmark execution using Darshan [26]. The input features with two categorised values are explicitly set to 0 and 1 as part of scaling. For example, the original possible values for I/O access pattern $(non-collective,collective)$ will be scaled to (0.0, 1.0). If categorised values are three as in case of parameter *unsorted order* in SEG-Y Sorting benchmarks, they are set to (0.0, 0.5, 1.0) against (*uniform*, *reverse*, *random*) values settings.

The Mean Square Error (MSE) value is computed at line 27 as the loss criterion set on line 16, to compute the loss in predicted bandwidth value of ANN’s feed forward propagation on line 25, in each iteration. The MSE values are computed using the following equation:

2 $$\begin{aligned} MSE = \frac{\sum _{i=0}^{n} (y_i - r_i)^2}{n} \end{aligned}$$

where $y_i$ is the predicted value of the model and $r_i$ is the real value of the $i^{th}$ test data from *n* number of test samples from benchmarks results. After computing the loss, the optimizer is being executed on line 29, to zero the gradients by applying the gradient descent method *Adam*, provided by PyTorch ANN’s API. After optimizing gradients, the loss is propagated backwards in ANN on line 31, followed by updating the parameters weights on line 33, accordingly. Then on line 34 *model.train()* finally updates itself with updated parameters weights.

Once the ANN models were trained enough, they were saved in their separate respective *ANN_MODEL.pt* files (on line 37). Those trained models were then applied to their respective test sets (20% of the complete data) of their benchmark results, with the intention of predicting I/O bandwidths on unseen data. The Section 4 shows the overall results of executed benchmarks and the prediction accuracy of the trained ANN based ML models.

### Prediction accuracy evaluation of ANN models

The prediction accuracy evaluation of ANN models is being conducted over the testing set of benchmarks results, the 20% of the complete data. This evaluation has been completed using the testing scheme mentioned under the later section of Prediction Results Analysis. The Listing 3 represents the overview of the code logic defined under *ANN_Model_Testing()* against the testing scheme. On line 5, the saved trained ANN model is being loaded from the file path *ANN_MODEL.pt*. Then its respective testing set is loaded in *X* and *y* containing scaled input feature values and output feature (I/O bandwidth) values respectively, on line 8 and 9. These values are scaled between 0 and 1 using *MaxAbsScaler* formula as mentioned earlier and the ANN models are trained on scaled values of their training sets. The value *n* on line 10 holds the total number of records or rows of the testing set.

Once the required model and its respective test set is loaded then model is being applied on test input feature configuration parameters values on line 13. The new predicted values are being stored in *y_predictions*. Then on line 16 and 17 the previous and predicted bandwidth values in *y* and *y_predictions* respectively, are re-scaled to their actual values by re-ordering Eq. 1 as following:

3 $$\begin{aligned} x = x' \times x_{max} \end{aligned}$$

where *x* represents initial or actual I/O bandwidth, $x'$ represents scaled bandwidth value and $x_{max}$ represents the maximum I/O bandwidth value recorded from the execution of a complete set of a particular benchmark type in Listing 3. After re-scaling actual bandwidth values the MAE, MSE, MAPE and Accuracy values are computed using the Equations 4, 2, 5 and 6 respectively, from line 20 to 26. Then finally mean accuracy values are further broken down into groups of different configuration sets within a test set of a benchmark types. All these errors and accuracy values are presented in Tables 4, 5, 6, 7,8.

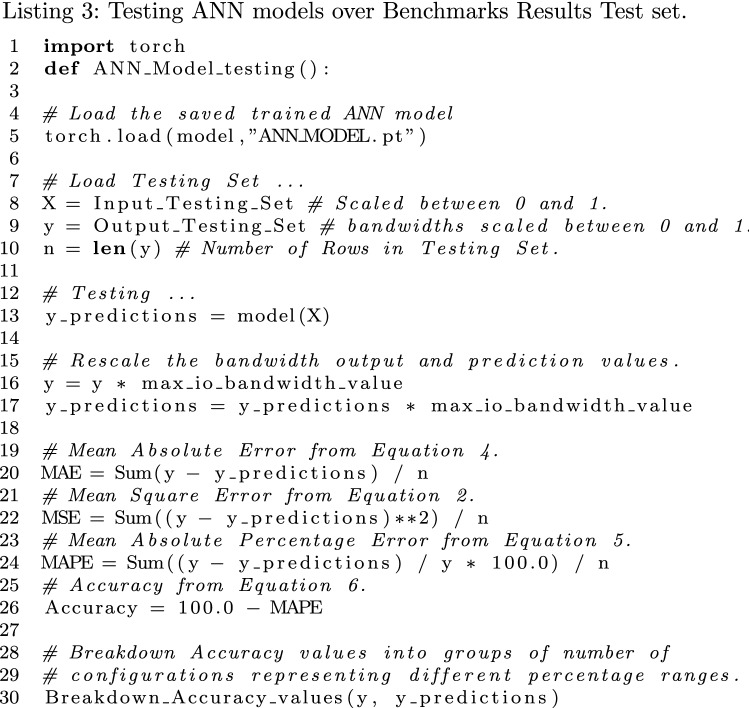



## Experimental result analysis

The Benchmarks have been executed on up-to 16 ICHEC’s KAY Cluster nodes [28] with each 2x 20-core 2.4 GHz Intel Xeon Gold 6148 (Skylake) processors employed on up-to 36 Lustre storage disks. The ML models have been trained and tested on KAY’s GPU node equipped with NVIDIA Tesla V100 16GB PCIe (Volta architecture) using PyTorch Tensors framework [16, 29].

### Benchmarks results

**Fig. 4:**
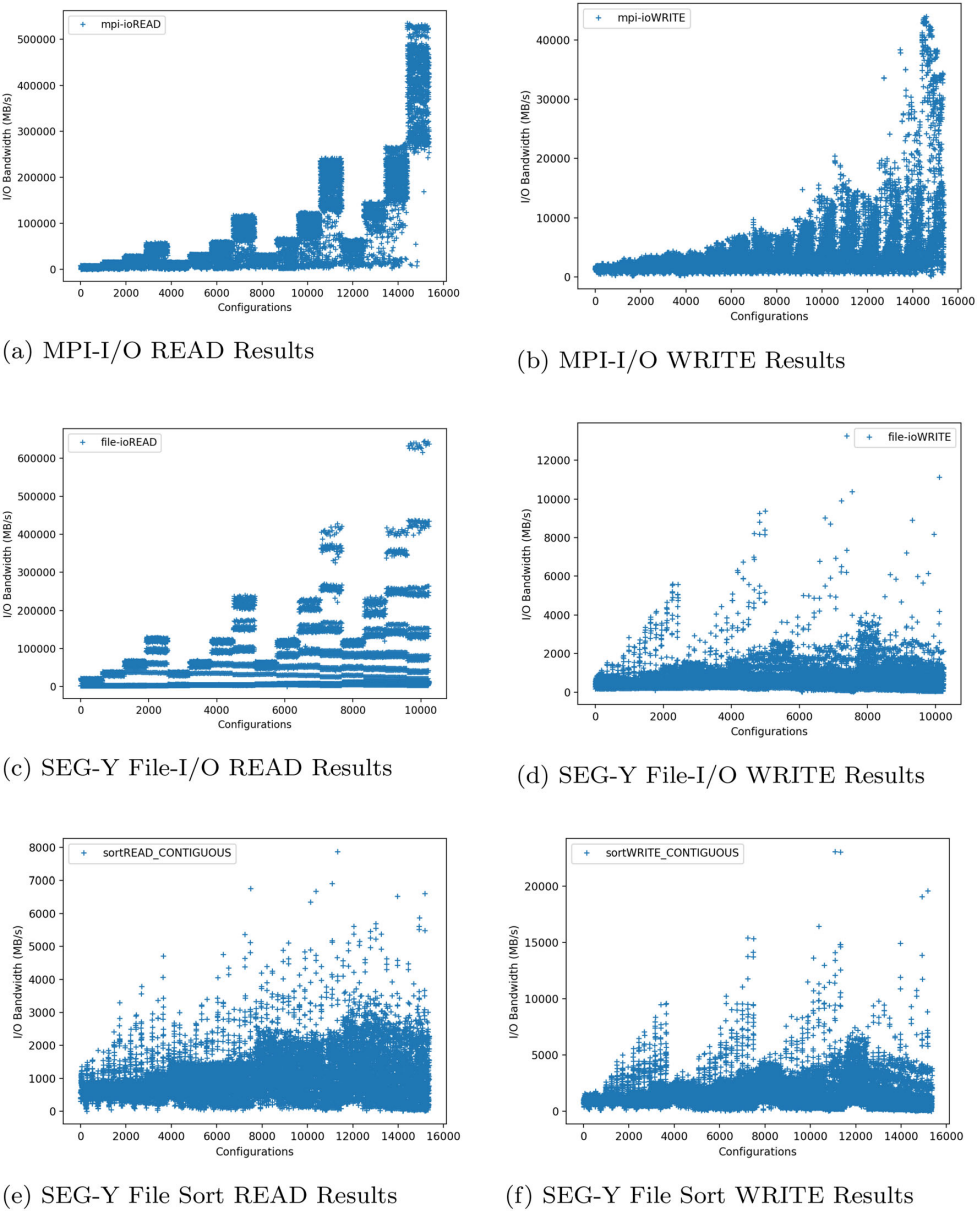
Benchmarks results graphs

The results graph are shown in Fig. 4 as scatter plots for both READ and WRITE operations for all benchmarks executed with same hierarchical order of nested loops as mentioned in Fig. 3. This HPC Cluster and its Lustre disks are not standalone and therefore data interference from other users is to be expected. Therefore, these benchmarks were executed over different periods of time repeatedly to ensure consistency. The x-axis on each graph represents the configuration number and y-axis represents the I/O bandwidth value in Mega Bytes per second (MB/s).

The configurations shown in Fig. 4, indicate how alternative configuration numbers shown on the x-axis, an associated bandwidth value (y-axis) can be established. The y-axis represents performance for a particular set of unique parameters which are not shown in the graphs. For this reason, it is not clear which parameter settings are causing certain I/O bandwidth performance values.

Therefore, all the following benchmark results were visualised in the parallel plot using HiPlot utility [23]. In particular, the high bandwidth configuration settings are highlighted.

The use of parallel plots in this context is novel and has not been utilised by papers that we have reviewed in this area. The evaluations explain the overall pattern of I/O behaviour, while also highlighting the specific configurations that exhibit high bandwidth scenarios.

#### MPI-I/O benchmarks evaluations

As mentioned before the possible combinations for basic MPI-I/O benchmark configurations are 30,720 (15,360 for READ and 15360 for WRITE separately). In relation to the READ bandwidth results, the benchmarks are executed with a common nested order given in Fig. 3 therefore, the outer loop executed for number of MPI nodes values. This means the first main groups of configurations are 4, corresponding to 4 values of number of MPI nodes in 1, in ascending order. These 4 groups of MPI nodes values can be seen in Fig. 4a. Each group is comprised of 4 steps of 960 configurations. These 4 steps in each MPI node value group corresponds to the MPI processes per node from Table 1 in ascending order, as the next Nested Loop 1 is executed according to Fig. 3. This means the steps in Fig. 4a are an indication that the overall I/O bandwidth increases as the MPI processes per node values also increase in each group of MPI nodes. As MPI nodes increase, the size of the steps also increases, which means the I/O bandwidth is significantly affected by the number of MPI nodes.

In WRITE bandwidth results the steps cannot be seen (in Fig. 4b) but the bandwidth is increasing as the overall number of MPI nodes and processes per node increase. The I/O (both READ and WRITE) performance is less impacted by the lustre striping parameter values as compared to the overall number of processes.

The data-aligning strategy suggested in [17–20] does not guarantee sufficient bandwidths where $MPI\_nodes < MPI\_processes\_per\_ node$. This means that more than one MPI process is running on each MPI node, which keeps the total number of MPI processes the same as in a case of $MPI\_nodes >= MPI\_processes\_per\_node$. The total number of MPI processes are equal to stripe count. Bandwidth can be worst affected with the collective I/O as compared to non-collective I/O.

**Fig. 5:**
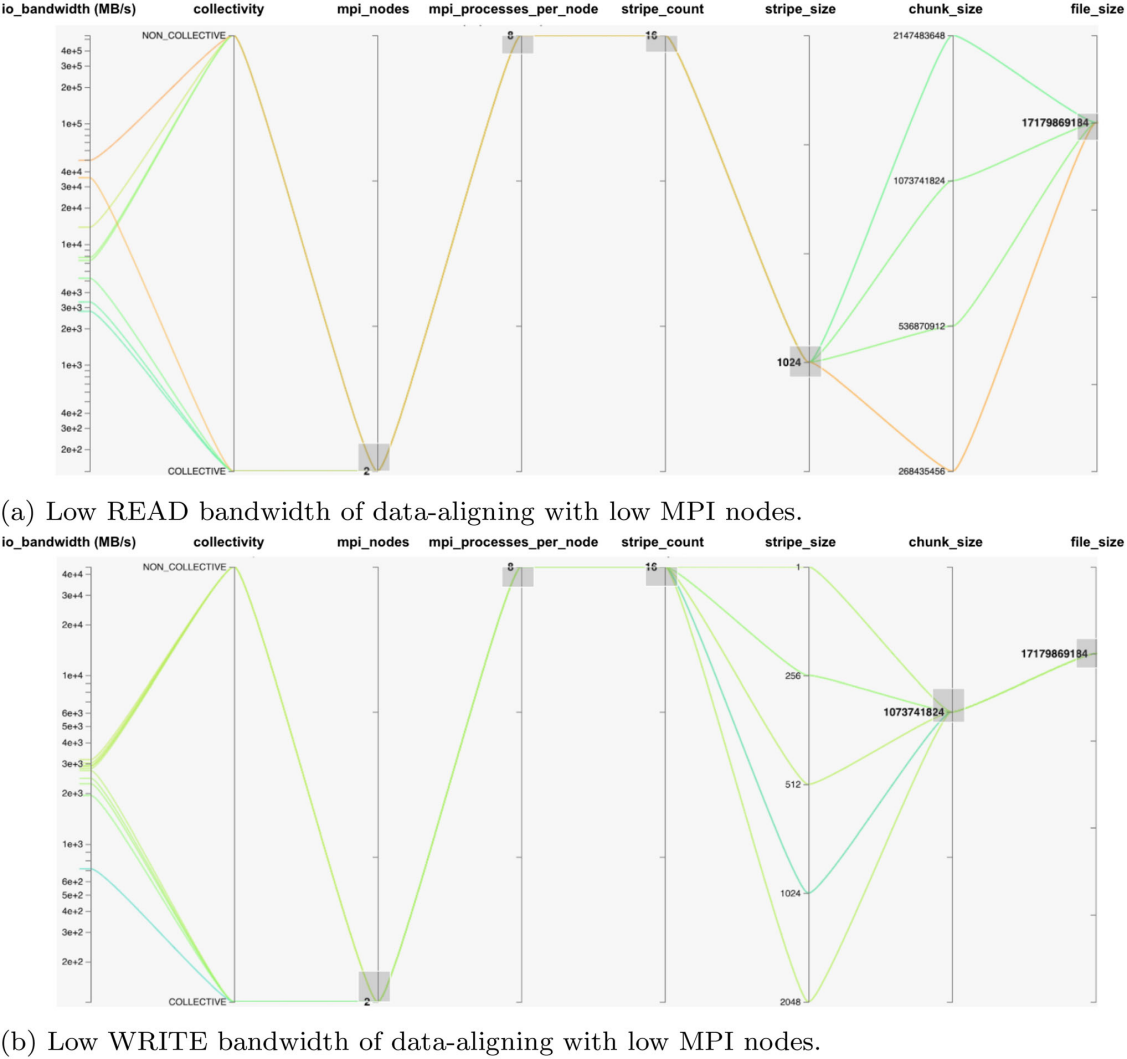
Data-aligning strategy showing Low I/O bandwidths. Note: stripe_size is represented in the units of Mega Bytes (MBs), chunk_size and file_size are represented in the units of Bytes

Figure 5a, b present an example scenario where 2 MPI nodes and 8 MPI processes per node (total 16 MPI processes) are reading and writing file data, respectively.

The low or decreased bandwidth values can be observed for both READ and WRITE operations when 16 MPI processes are aligned with 16 lustre disks (OSTs stripe count value) and stripe size is aligned with chunk size, completing the data-aligning strategy.

These figures also suggest the settings to overcome this problem where stripe size is not aligned with chunk size, in both READ and WRITE operations scenarios. For READ operation in Fig. 5a lowering the chunk size per MPI processes improves the bandwidth. The minimum chunk size gives the highest bandwidth for both collective and non-collective READ operations.

Changing stripe size in this scenario is not considered because it has no benefit at runtime. In this case, the file is already distributed and striped across the disks with certain striping unit (size). The different bandwidth values for different stripe sizes also exists, provided that the file is already generated with the same stripe size.

For WRITE operations in Fig. 5b, changing or lowering the stripe size doubles the bandwidth from the data-aligned parameters in this case. This jump in bandwidth may not always be the case as the disks are not standalone, due to the continuous interference from other users I/O processes on the HPC cluster. However, the I/O bandwidth still can be increased with some difference, possibly from the lowest stripe size value (1 MB), as shown in this scenario. The other chunk size values are not shown but they can also make small differences in increasing the WRTIE operation bandwidth.

#### SEG-Y File I/O benchmarks evaluations

For SEG-Y Files benchmarks, READ operations graph (in Fig. 4c) somehow shows a similar pattern as in simple MPI-I/O READ operations in Fig. 4a. Considering the same nested loop patterns in Fig. 3, for each number of MPI node values, the bandwidths steps increase as the value of MPI processes per node increases. As the value of MPI nodes increases the size of the bandwidth step also increases.

**Fig. 6:**
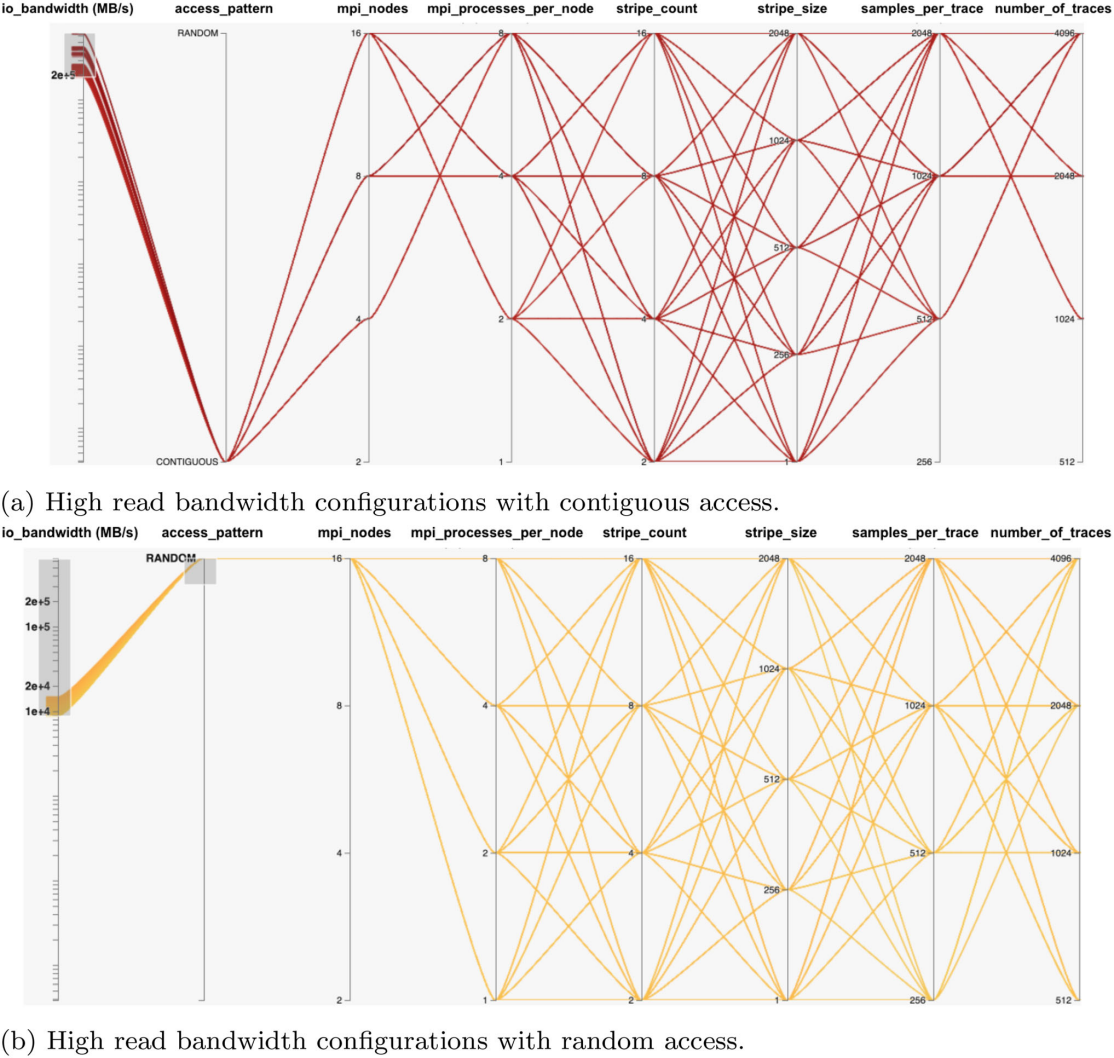
High bandwidth configurations settings for SEG-Y File READ operations. Note: stripe_size is represented in the units of Mega Bytes (MBs)

In the case of when the threshold is set at 200000 (MB/s), fewer combinations are observed above this value. When visualized in HiPlot, these configurations have higher MPI process values, higher samples per trace, and higher number of traces values for contiguous READ. This is visible in Fig. 6a. If random READ is to be visualised, then Fig. 6b shows the highest MPI nodes value is required for most of the cases. The SEG-Y File WRITE operations graph in Fig. 4d are different from basic MPI-I/O WRITE operations in Fig. 4b. The reason for this, is the parameters involved in SEG-Y File WRITE are different from the basic MPI-I/O WRITE operation.

Keeping the SEG-Y File structure (Fig. 4d) in view, each MPI process would be interested in writing specific parts of the SEG-Y File. In particular, those parts are trace data which follows trace headers of 240 bytes. Normally a chunk size is not known to a process which can be aligned with stripe size. The other factor is the access pattern which can be contiguous or random. But as the striping occurs when writing a file on a parallel file system (LFS), the striping parameters still have some impact on bandwidth.

**Fig. 7:**
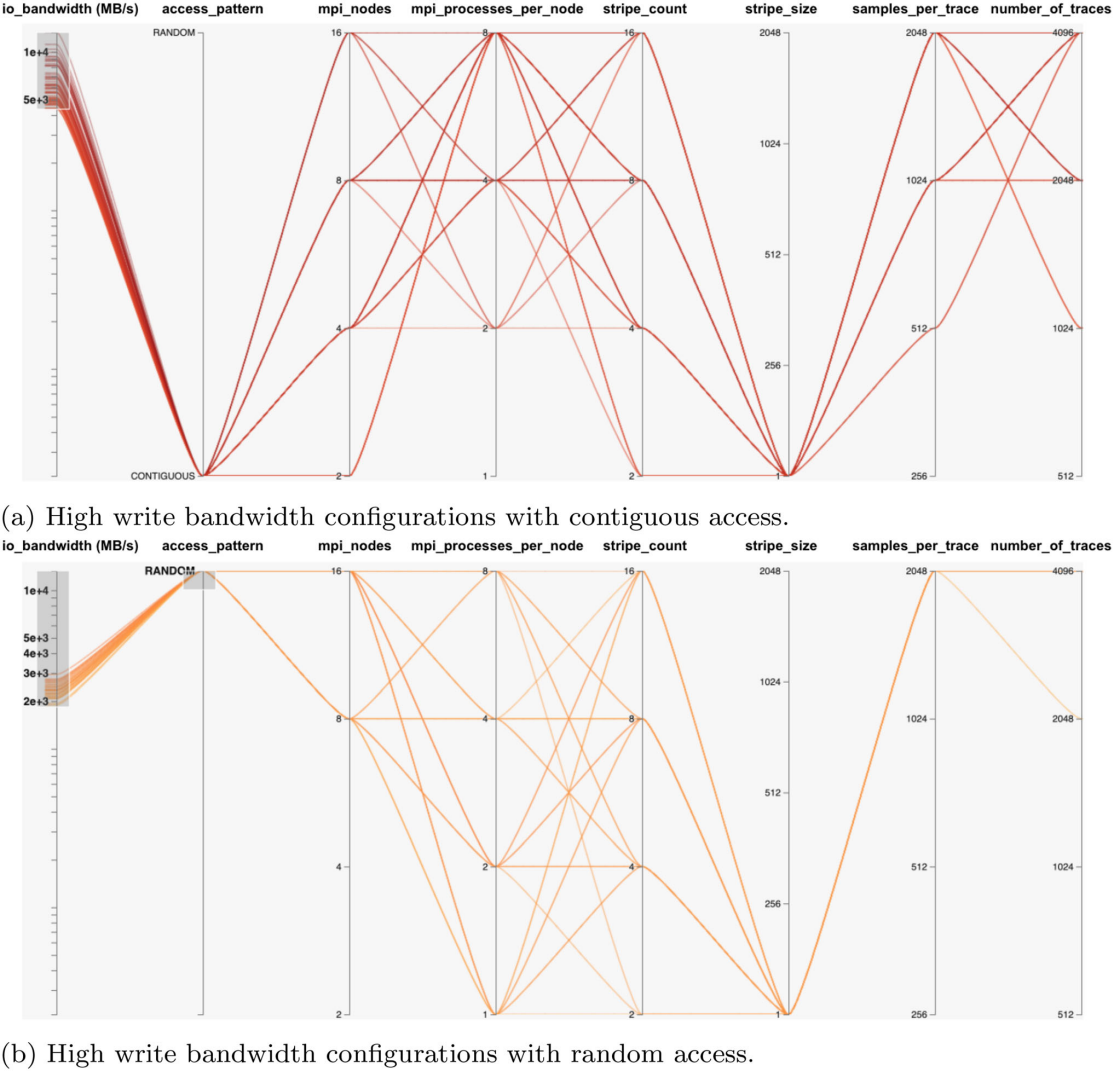
High bandwidth configurations settings for SEG-Y File WRITE operations. **Note:-** stripe_size is represented in the units of Mega Bytes (MBs)

In Fig. 4d, the rising peaks can be seen indicating that for each MPI nodes value group, but overall there are numerous low bandwidth configurations, and fewer high bandwidth configurations. It is easier to identify those configuration settings with high or rising bandwidth values. The low bandwidth scenarios occur from value 4000 (MB/s) and below whereas higher ones occur at greater values. The Fig. 7a shows those parameter values and their combinations. It can be seen that 1 MB stripe size (regardless of stripe count), higher samples per trace, number of traces and overall number of MPI processes using contiguous pattern gives higher SEG-Y File WRITE bandwidth values. If random pattern is to be visualised for high bandwidth configuration settings then Fig. 7b shows the 1 MB stripe size with highest samples per trace, number of traces and MPI nodes values. The number of traces and samples per trace values ultimately define the size of the SEG-Y File. This also means the large SEG-Y File is more likely to give improved WRITE bandwidth as compared to smaller file sizes.

#### SEG-Y file sorting benchmarks evaluations

We now examine benchmarks relating to SEG-Y File Sorting. These benchmarks read or write a file contiguously that was previously generated using any of these unsorted orders: uniform (ascending order), random and uniform reverse (descending order) with respect to x-source of trace header value in the file.

It is important to then determine which combinations of configuration parameters values give good or high I/O bandwidth values for both reading and writing a file contiguously with different unsorted orders. Both results in Fig. 4e, f show numerous low bandwidth plots and fewer high bandwidth plots. It is again important to identify the combinations of configurations settings with high bandwidths after certain threshold values, as in the case of SEG-Y File-I/O benchmarks findings.

**Fig. 8:**
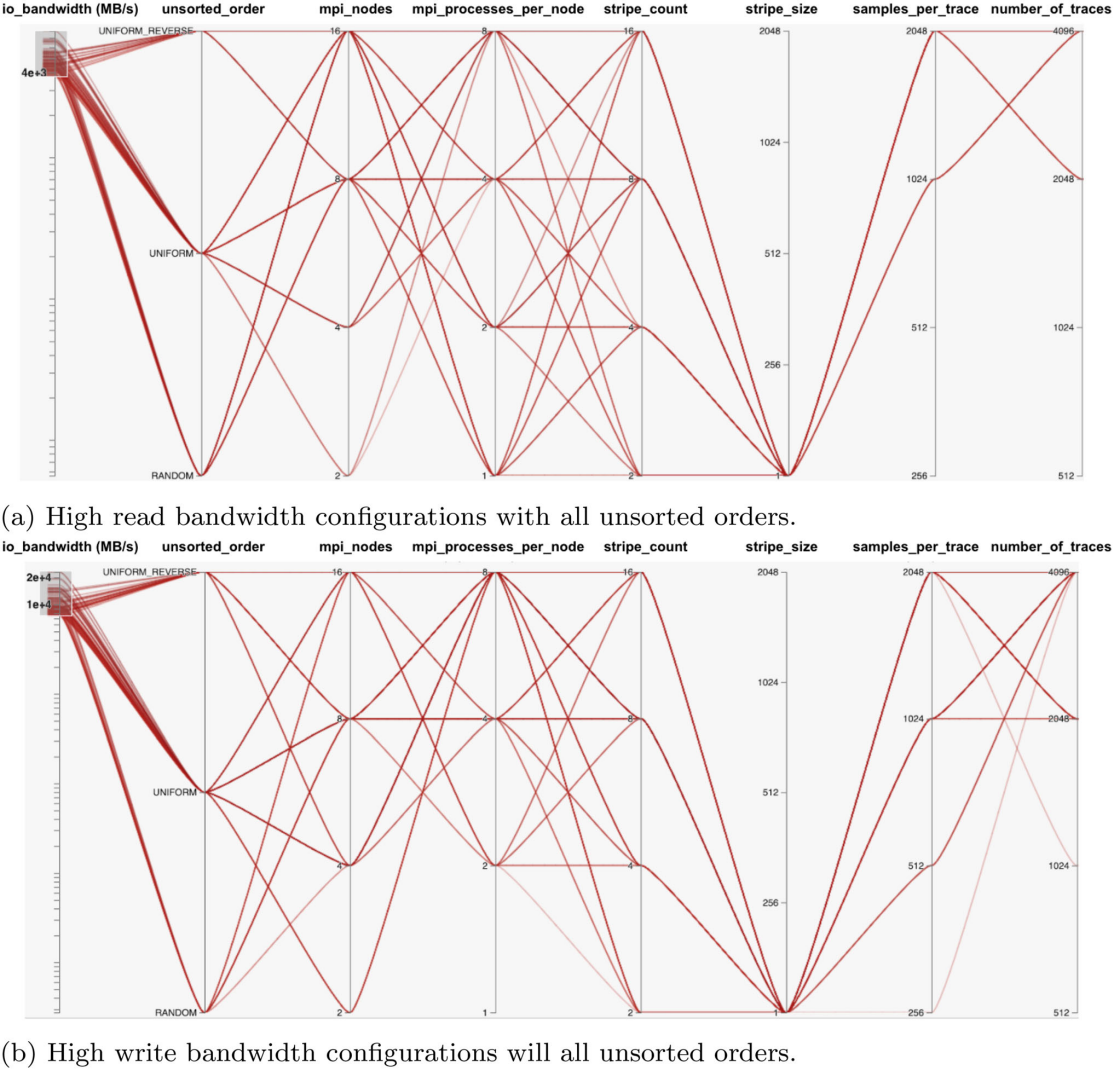
High bandwidth configurations settings for SEG-Y File Sorting. Note: stripe_size is represented in the units of Mega Bytes (MBs)

First, considering the READ operation results shown in Fig. 4e, the highest bandwidth plots occur from 4000 (MB/s), which represents the upper half of the graph. Setting those values in the HiPlot utility gives us an insight of all possible combinations in Fig. 8a. The settings shown are for each unsorted order, have a high total number of MPI processes, and regardless of stripe count, the stripe size should be set to 1MB, and the number of traces with samples per trace should be as high as possible. These configuration settings are shown to yield high READ bandwidths.

With respect to the WRITE operation results in Fig. 4f, the highest bandwidths plots occur from some values greater than 10000 (MB/s) and above. This is again the upper half of the graph as it was in READ operation results mentioned previously. A visualization of these bandwidth values and combinations in HiPlot is shown in Fig. 8b. The settings shown are for each unsorted order, have a high total number of MPI processes, and regardless of stripe count, the stripe size should be set to 1MB, and the number of traces with samples per trace should be as high as possible. These configuration settings are shown to yield high WRITE bandwidths. It is worth noting that the approach identified between READ and WRITE operation results was identical.

The data shown in this section has demonstrated that there are no continuously increasing or continuously decreasing patterns in the data that can be assumed. Instead, we notice, changing patterns of I/O bandwidth over alternative configurations of parameter values.

It has been shown that most configurations result in low bandwidth values while only a small number result in more optimal higher bandwidth values. Therefore, it demonstrates the need for an approach that will identify good combinations of settings, which can achieve better overall performance. The challenge is to identify these combinations of settings prior to the execution of any I/O operations begin.

For this reason, our research has proposed to apply Neural Networks as a means of making certain predictions about the optimal combinations of settings. Neural Networks are an appropriate approach to allow the system to learn these changing patterns over different features or parameters which effect the I/O bandwidth performance so significantly.

### Prediction results anaysis

The ANN models for all the benchmarks have been applied to their respective test sets as means of prediction. The MPI-I/O and SEG-Y Sorting benchmarks have 3072 number of configurations tested for each of their READ and WRITE operations. Whereas the SEG-Y I/O benchmarks have 2048 tested configurations for each READ and WRITE operation.

#### Testing scheme

The prediction results of our ANN models on their related test sets are determined via different accuracy metrics, namely: MSE (Mean Squared Error) values, MAPE (Mean Absolute Percentage Error), percentage accuracy and MAE (Mean Absolute error) [30–33]. This was done to check the accuracy of bandwidth predictions that how fit is our model as compare to bandwidth pattern generated by benchmarks results in Fig. 4. The prediction percentage accuracy has also been used as a metric for measuring the precision of ML models in couple of previous research works [10, 12, 15]. The Table 4, shows the computed MSE values during training and testing, and MAE in I/O bandwidth prediction on test set, for each benchmarks READ and WRITE operations models. The MAE in 5$^{th}$ column of this table, MAPE and percentage accuracy in Table 5, are computed using the following equations:

4 $$\begin{aligned}&MAE = \frac{\sum _{i=0}^{n} (y_i - r_i)}{n} \end{aligned}$$

5 $$\begin{aligned}&MAPE = \frac{1}{n} \sum _{i=0}^{n} \Bigg |\frac{(y_i - r_i)}{y_i}\Bigg |\times 100.0\% \end{aligned}$$

6 $$\begin{aligned}&Accuracy = 100.0 - MAPE \end{aligned}$$

where $y_i$ is the predicted value of the model and $r_i$ is the real value of the $i^{th}$ test data from *n* number of samples in test set from benchmarks results data.

**Table 4:** Errors during training and testing

Benchmark	I/O	MSE (training)	MSE (testing)	MAE (MB/s)
MPI-I/O	READ	6.09e-4	6.66e-4	7530.36
	WRITE	4.05e-3	5.04e-3	1244.14
SEG-Y I/O	READ	1.04e-6	2.07e-5	1111.05
	WRITE	8.60e-5	1.65e-4	82.34
SEG-Y Sort	READ	7.28e-4	9.99e-4	167.82
	WRITE	1.98e-4	3.59e-4	249.45

**Fig. 9:**
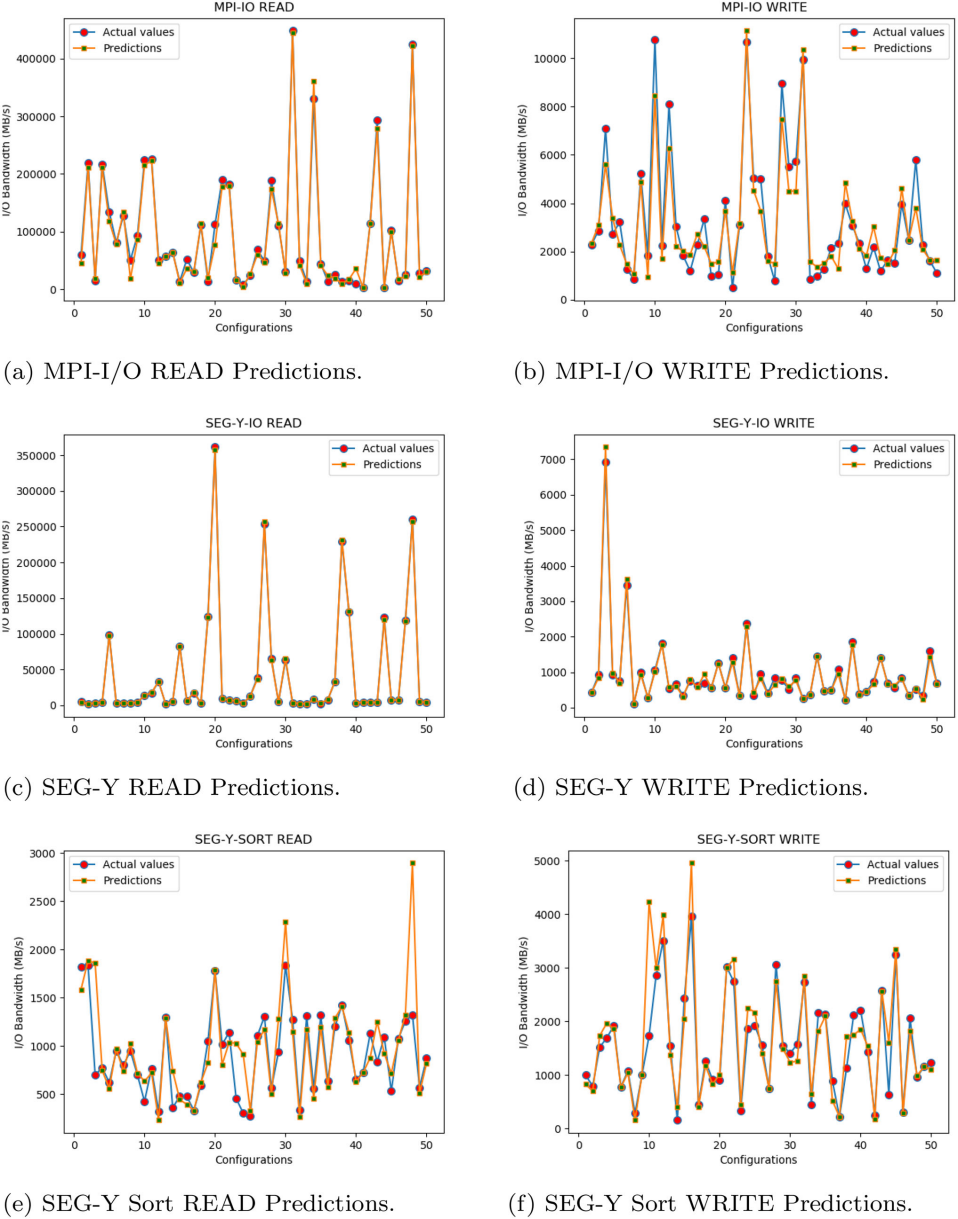
Predictions results using trained ANN models

In Table 4, the MSE values presented after our final testing shown in 4$^{th}$ column are closer to zero and slightly greater than the MSE of training in the 3$^{rd}$ column. This indicates that the trained ANN models for each benchmark type are not under-fitted and very less over-fitted according to [34], which is further supported by Fig. 9. The MAE values in 5$^{th}$ column represents the mean difference of predicted I/O bandwidth values from the actual I/O bandwidth values of benchmarks results. These MAE values in predictions are very small in comparison to the range or scale of actual I/O bandwidth values given on the y-axis of benchmarks graphs in Fig. 4. As evident from the table, the MPI-I/O READ’s ANN model gives the highest MAE value despite the fact it mostly follows the pattern similar to actual bandwidth values as shown in Fig. 9a.

#### Testing results analysis

The Fig. 9 presents 50 randomly selected configurations from the test set with their actual and predicted I/O bandwidth values for each model of a benchmark type. It demonstrates that the models can predict the I/O bandwidth values according to the pattern or behaviour exhibited by actual values from the benchmarks results in Fig. 4, for future unseen configuration data.

The accuracy ranges from 62.5 to 96.5% with respect to our models, as shown in Table 5 - 4$^{th}$ column. This results from the MAPE values ranging from 3.5% to 37.5%, in the 3$^{rd}$ column. There are few cases while making predictions on test set, where predicted bandwidth is larger than the actual bandwidth value, results in negative percentage accuracy values. This affects the overall accuracy of a model but still prediction follows the right pattern in estimating bandwidth, as shown in Fig. 9. Excluding these cases results in increased accuracy values as mentioned in 5$^{th}$ column. It is exception to the case of SEG-Y I/O READ ANN model with the highest accuracy among all the models, which has no case of negative accuracy values when predicting I/O bandwidth on its test set configurations.

**Table 5:** Accuracy of applied ANN models

Benchmark	I/O	MAPE	Accuracy (%)	Accuracy without -ve values (%)
MPI-I/O	READ	37.5	62.5	83.5
	WRITE	27.9	72.1	76.3
SEG-Y I/O	READ	3.5	96.5	96.5
	WRITE	11.9	88.1	90.4
SEG-Y Sort	READ	23.0	77.0	84.8
	WRITE	20.0	80.0	85.2

The mean accuracy values are further breakdown in different number of configuration cases of test set in Tables 6, 7 and 8. The 1$^{st}$ and 3$^{rd}$ columns of these tables represents the 11 different groups of number of configurations from the test set. Each group has its own mean accuracy percentage. For example, in Table 6, a group of 484 READ Test configurations have their I/O bandwidth predicted with 85.7% Mean Accuracy. This is exception to the case of SEG-Y I/O READ tests have few groups as almost all test configurations covered and resulted in the high accuracy values groups . The 2$^{nd}$ and 4$^{th}$ column represents their respective mean accuracy values. This shows that most of the predictions on test set cases tend towards the high percentage accuracy values. There are few with very low or negative mean accuracy values. The Table 6 shows $\approx$7% and $\approx$3% of the READ and WRITE test configurations predicted with negative mean accuracy values, respectively, shown in the second last row. Similarly, the Table 7 shows 0% and $\approx$1%, and Table 8 shows $\approx$4% and $\approx$3%, of their READ and WRITE test configurations, respectively, being predicted with negative mean accuracy values. Dividing the sum of all Mean Accuracy values by total tests, for each benchmark type, results in overall accuracy of a model as represented in the 4$^{th}$ column of Table 5. Excluding the negative accuracy values and the corresponding test cases from them, results in overall accuracy without negative values, as represented in in the 5$^{th}$ column of Table 5.

**Table 6:** Accuracy breakdown for MPI-I/O benchmarks prediction models

READ tests	Mean accuracy (%)	WRITE tests	Mean accuracy (%)
1580	96.0	794	95.1
484	85.7	726	85.1
273	75.5	612	75.2
192	64.9	358	65.5
99	54.8	216	55.4
74	45.3	109	45.5
54	36.3	69	35.5
39	25.5	50	25.4
28	15.0	33	15.0
36	5.6	22	5.3
213 ($\approx$7%)	-218.2	83 ($\approx$3%)	-79.4
Total=3072		Total=3072

**Table 7:** Accuracy breakdown for SEG-Y I/O benchmarks prediction models

READ tests	Mean accuracy (%)	WRITE tests	Mean accuracy (%)
1907	97.4	1371	95.5
118	86.7	444	86.1
18	76.6	120	76.0
4	67.0	48	65.1
1	44.9	21	54.4
-	-	9	46.0
-	-	3	34.5
-	-	2	21.9
-	-	4	13.3
-	-	3	5.1
- (0%)	-	23 ($\approx$1%)	-122.8
Total=2048		Total=2048

**Table 8:** Accuracy breakdown for SEG-Y Sorting benchmarks prediction models

READ tests	Mean accuracy (%)	WRITE tests	Mean accuracy (%)
1338	95.2	1431	95.1
919	85.6	896	85.8
381	75.9	323	75.9
132	66.1	131	65.8
69	55.6	60	55.7
31	44.4	42	44.4
34	35.1	30	36.0
24	24.7	17	23.7
21	14.5	22	15.1
12	5.4	20	6.3
111 ($\approx$4%)	-129.1	100 ($\approx$3%)	-75.6
Total=3072		Total=3072	

An important consideration is that the trained ANN models are following almost the same trend when predicting the I/O bandwidth values on different sets of configurations, as compared to actual benchmarks. It depicts that if one configuration has greater I/O bandwidth than the other configuration then, the same behaviour is expected by the prediction model.

Since the trained ANN models are simulating the pattern of changing I/O behaviour, their usefulness is to compare the bandwidth values among the different configurations. This feature of our ML models can be used to detect and tune the best configuration settings for any given scenario, prior to the execution of I/O processing in the MPI application. This is hugely significant, in the ongoing challenge of more efficiently processing seismic data through SEG-Y Files.

## Discussion

In the previous results section, we have presented a series of experiments. These show that certain configuration parameters are involved in setting high or low I/O bandwidths i.e. the MPI processes, lustre striping parameters, size of trace data in SEG-Y file, etc. This information assisted in identifying the exact configuration parameters for our basic MPI-I/O file operations and SEG-Y File I/O/sorting operations in ExSeisDat.

As per related work, the common practice to improve HPC-I/O in different scenarios is the prediction of I/O bandwidth over the configuration parameters which can be tuned beforehand to give the improved bandwidth afterwards. We note that, perceiving the changing behaviour of I/O bandwidth performance is difficult when multiple configurations are involved. We determined that the best approach for prediction was an ML approach based on ANNs, due to their high accuracy [12, 14, 15].

Once the key parameters were identified, we conducted benchmarks on the generated list of all possible configurations, to provide training and testing data to our ML models. As the SEG-Y File I/O and Sorting are common operations in ExSeisDat apart from basic MPI-I/O File operations, we categorised the benchmarks into three types; (1) MPI-I/O benchmarks for basic file read and write operations, (2) SEG-Y File I/O benchmarks and (3) SEG-Y File Sorting benchmarks. Our findings on each benchmark, explains the I/O behaviour. This is shown by our graphs in Fig. 4 and highlights the high bandwidth configurations on parallel plots using HiPlot Utility in Figs. 5, 6, 7 and 8, which has not previously been presented by any state-of-the-art research.

In the case of the remaining configurations with low bandwidths, as shown in section of benchmarks results in Fig. 4. These were greater in number, and therefore it is difficult to determine the I/O pattern that each configuration contributes to increased or decreased performance. This results in our development of the ML ANN models for each benchmark type to predict their I/O bandwidth over different configurations. The prediction models are trained and tested through different metrics such as MSE, MAE and Accuracy using MAPE, values. The models learned the trend of changing I/O behaviour which is reflected in the prediction results graphs shown in Fig. 9. As has been shown, the prediction models can predict the I/O bandwidth according to the learned trend for future unseen configurations. Therefore, these are hugely beneficial in tuning the configuration parameters for basic MPI-I/O operations and SEG-Y operations in ExSeisDat.

Having a well-trained model, the execution path or flow can be completed from prediction to tuning parameters in application before I/O operations. The steps of execution can be: (1) Get the current set of configuration values, (2) predict its I/O bandwidth (2) Check different parameters settings and compare their prediction bandwidths values with current predicted value, (3) Choose the settings predicting maximum I/O bandwidth value and (4) Tune the configuration parameters with chosen settings. Therefore, it is possible that current or existing parameter settings predict maximum bandwidth out of all other possible settings being checked, and parameters may not be tuned to different values. In relation to tuning parameters on runtime execution of MPI program, it should be noted that not all the parameters are tunable except some of them. For example, in SEG-Y WRITE operation the access pattern, stripe count and stripe size values can be tuned but MPI processes and processes per node cannot be changed as the MPI application is already in execution, and number of traces and samples per trace values cannot be changed being the requirement of a user. Therefore, a set of tunable parameters values should be chosen, predicting higher or maximum I/O bandwidth with the given non-tunable parameters values. This also means tuning parameters logic will be different with respect to type of operation. Despite this, eventually the tuning parameters based on the ML prediction can be gainful in a longer term due to forecasting bandwidth beforehand and adapting the optimized configurations.

## Conclusion

In this paper, we examined how MPI-I/O performance varies in ExSeisDat over SEG-Y files on LFS [3, 4, 6]. The paper first demonstrates the need for a flexible and efficient optimisation approach through a series of experimental benchmarks. This is similar to much existing research which examines how I/O performance can prove challenging in these scenarios [7–12]. There are two key contributions outlined in this paper. Firstly, the provision of parallel plots that give clear and succinct insights into the high bandwidth performance of the system across various configuration parameters. Secondly, the application of the ANN based ML approach to the prediction of I/O bandwidth using the previously identified benchmarks. The prediction accuracy ranges from 62.5% to 96.5% throughout the trained ANN models for those benchmarks. Previous studies in this area which have been reviewed earlier in this paper, indicate the limitations of previous approaches to each of these contributions. Our results show that these contributions have a significant benefit to practitioners in this field, by contributing major improvements on overall bandwidth prediction, which in itself has many other knock-on benefits with respect to parameter tuning. Thereby, improving the overall efficient completion of tasks for seismic data processing.

## Data Availability

The benchmarks data sets generated and used in this research are available on request.
